# Feature tracking CMR reveals abnormal strain in preclinical arrhythmogenic right ventricular dysplasia/ cardiomyopathy: a multisoftware feasibility and clinical implementation study

**DOI:** 10.1186/s12968-017-0380-4

**Published:** 2017-09-01

**Authors:** Mimount Bourfiss, Davis M. Vigneault, Mounes Aliyari Ghasebeh, Brittney Murray, Cynthia A. James, Crystal Tichnell, Firdaus A. Mohamed Hoesein, Stefan L. Zimmerman, Ihab R. Kamel, Hugh Calkins, Harikrishna Tandri, Birgitta K. Velthuis, David A. Bluemke, Anneline S. J. M. te Riele

**Affiliations:** 1 Radiology and Imaging Sciences, National Institutes of Health Clinical Center, Bethesda, MD USA; 2 Department of Medicine, Division of Cardiology, University Medical Center Utrecht, Utrecht, the Netherlands; 3 Department of Engineering Science, Institute of Biomedical Engineering, University of Oxford, Headington, Oxford, UK; 4 Sackler School of Graduate Biomedical Sciences, Tufts University School of Medicine, Boston, MA USA; 5 Department of Radiology, Johns Hopkins Hospital, Baltimore, MD USA; 6 Department of Medicine, Division of Cardiology, Johns Hopkins Hospital, Baltimore, MD USA; 7 Department of Radiology, University Medical Center Utrecht, Utrecht, the Netherlands; 8 Netherlands Heart Institute, Utrecht, the Netherlands

**Keywords:** Feature tracking cardiac magnetic resonance imaging, Regional myocardial strain, Global myocardial strain, Software comparison study, Arrhythmogenic right ventricular dysplasia/Cardiomyopathy

## Abstract

**Background:**

Regional right ventricular (RV) dysfunction is the hallmark of Arrhythmogenic Right Ventricular Dysplasia/Cardiomyopathy (ARVD/C), but is currently only qualitatively evaluated in the clinical setting. Feature Tracking Cardiovascular Magnetic Resonance (FT-CMR) is a novel quantitative method that uses cine CMR to calculate strain values. However, most prior FT-CMR studies in ARVD/C have focused on global RV strain using different software methods, complicating implementation of FT-CMR in clinical practice. We aimed to assess the clinical value of global and regional strain using FT-CMR in ARVD/C and to determine differences between commercially available FT-CMR software packages.

**Methods:**

We analyzed cine CMR images of 110 subjects (39 overt ARVD/C [mutation+/phenotype+], 40 preclinical ARVD/C [mutation+/phenotype-] and 31 control) for global and regional (subtricuspid, anterior, apical) RV strain in the horizontal longitudinal axis using four FT-CMR software methods (Multimodality Tissue Tracking, TomTec, Medis and Circle Cardiovascular Imaging). Intersoftware agreement was assessed using Bland Altman plots.

**Results:**

For global strain, all methods showed reduced strain in overt ARVD/C patients compared to control subjects (*p* < 0.041), whereas none distinguished preclinical from control subjects (*p* > 0.275).

For regional strain, overt ARVD/C patients showed reduced strain compared to control subjects in all segments which reached statistical significance in the subtricuspid region for all software methods (*p* < 0.037), in the anterior wall for two methods (*p* < 0.005) and in the apex for one method (*p* = 0.012). Preclinical subjects showed abnormal subtricuspid strain compared to control subjects using one of the software methods (*p* = 0.009). Agreement between software methods for absolute strain values was low (Intraclass Correlation Coefficient = 0.373).

**Conclusions:**

Despite large intersoftware variability of FT-CMR derived strain values, all four software methods distinguished overt ARVD/C patients from control subjects by both global and subtricuspid strain values. In the subtricuspid region, one software package distinguished preclinical from control subjects, suggesting the potential to identify early ARVD/C prior to overt disease expression.

**Electronic supplementary material:**

The online version of this article (10.1186/s12968-017-0380-4) contains supplementary material, which is available to authorized users.

## Background

Feature Tracking Cardiovascular Magnetic Resonance (FT-CMR) is a rapidly emerging approach for the quantitative and noninvasive evaluation of regional myocardial function. It employs a frame-to-frame recognition of a preset feature during the cardiac cycle, which allows for the calculation of myocardial displacement during systole expressed in strain values [1, 2]. Compared to other strain analysis techniques, e.g. CMR tissue tagging and echocardiographic speckle tracking, FT-CMR has shorter post-processing times, may be less operator dependent, and can be applied to routine cine CMR images [3]. In addition, FT-CMR has major advances over other deformation techniques in the evaluation of the right ventricle (RV), since it allows for reliable tracking of the highly trabeculated and thin walled RV and is not hampered by the anatomic localization of the RV behind the sternum [4, 5]. As such, FT-CMR may play an important role in the evaluation of diseases affecting the RV.

Arrhythmogenic right ventricular dysplasia/cardiomyopathy (ARVD/C) is an inherited cardiomyopathy that primarily affects RV morphology and function [6]. Since one of the most feared disease presentations (especially in the young and in athletes) is sudden cardiac death, early diagnosis is of utmost importance [7, 8]. One of the hallmarks of ARVD/C is regional dysfunction of the RV wall [9]. However, most prior studies have focused on evaluation of global RV strain in clinically overt ARVD/C patients [10–12]. We hypothesize that FT-CMR may be useful for early disease detection in ARVD/C by identifying regional myocardial dysfunction prior to overt disease development.

FT-CMR of the RV is relatively new, and early results have shown the feasibility of the method in ARVD/C [12–14]. For clinical implementation, it is important that FT-CMR is reproducible and that different software methods provide comparable strain values. We therefore aimed to 1) assess intersoftware agreement of RV global and regional longitudinal strain using FT-CMR; and 2) compare global and regional strain in definite ARVD/C patients, preclinical ARVD/C subjects and control subjects to analyze the value of regional strain as an early diagnostic parameter. To accomplish this, we used a unique cohort of well-phenotyped ARVD/C subjects that includes both affected patients and at-risk mutation carriers.

## Methods

### Study population

We included 110 subjects who were evaluated for ARVD/C at the Johns Hopkins Hospital and were included in the Johns Hopkins ARVD/C registry (*ARVD.com*). Cases included 79 ARVD/C-associated desmosomal mutation carriers who were divided in two groups: 1) overt ARVD/C (those fulfilling 2010 diagnostic Task Force Criteria [TFC] for ARVD/C, *n* = 39); and 2) preclinical ARVD/C (those not fulfilling 2010 diagnostic TFC for ARVD/C, *n* = 40) [15]. All overt patients were diagnosed with ARVD/C independent of CMR, so that the diagnostic TFC provide an independent standard of reference. As a control group, we included 31 individuals who were mutation-negative family members of mutation-positive ARVD/C patients (*n* = 9), or subjects without ARVD/C upon comprehensive clinical evaluation (*n* = 22). All subjects were also included in a prior study from our group [13]. Patients provided written informed consent, and the study protocol was approved by the Johns Hopkins School of Medicine Institutional Review Board.

### CMR acquisition

All CMR images were acquired on a 1.5 Tesla scanner (Avanto, Siemens Medical Imaging, Erlangen, Germany) using a cine balanced, steady state free precession sequence (repetition time/echo time/flip angle −2.4/1.2/50–75 degrees, matrix 256–192, field of view 30-36 cm, temporal resolution ≤40 msec, slice thickness 6-8 mm).

### FT-CMR software methods

Peak longitudinal strain measurements were performed using four different commercially available FT-CMR software methods: 1) Medis Qstrain Software (Medis Medical Imaging Systems, version 2.1.12.2. Leiden, the Netherlands); 2) TomTec (TomTec Imaging Systems, version 2D CPA MR 1.2. Unterschleissheim, Germany); 3) Multimodality Tissue Tracking (MTT) (Toshiba Medical Systems Corporation, version 6.0.4725. Tokyo, Japan); and 4) Circle Cardiovascular Imaging (CVI42, version 5.6.2. Calgary, Canada). The most recent software versions available at time of measurement were used.

### Quantitative analysis

#### Myocardial strain analysis using FT-CMR

**Fig. 1:**
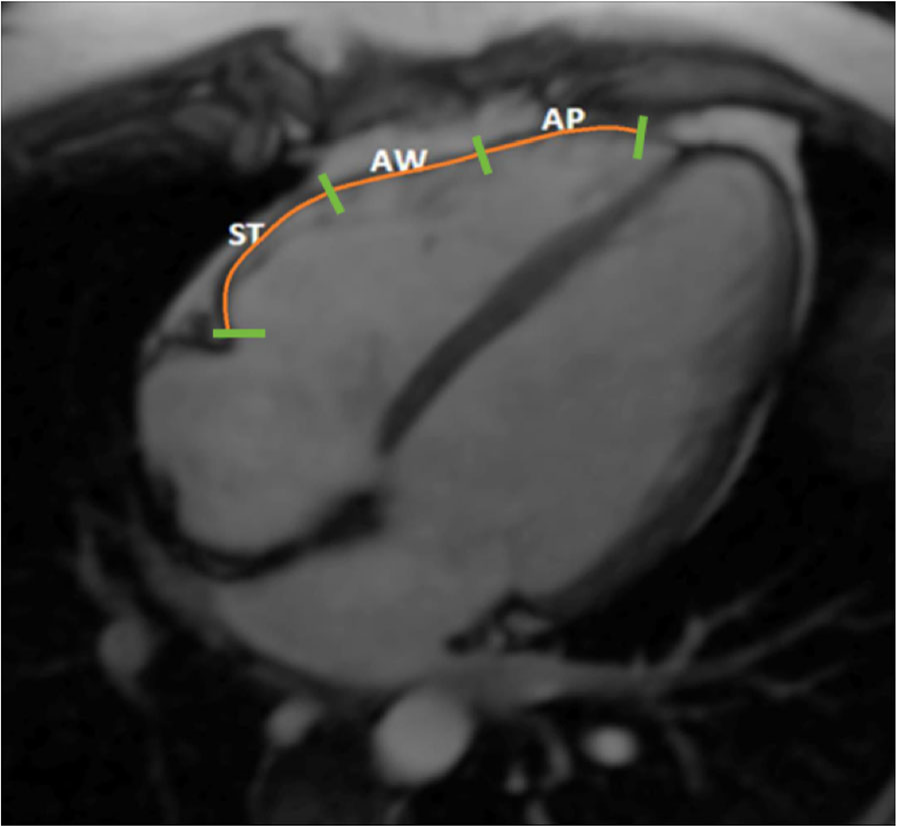
Representative right ventricular segmentation used in Feature Tracking Cardiac Magnetic Resonance Imaging. Abbreviations: ST = subtricuspid region; AW = anterior wall region; AP = apical region

Since previous studies have shown that wall motion abnormalities in ARVD/C are most reliably measured in the horizontal long axis (HLA, i.e. four chamber view), we used this view to determine peak longitudinal strain as a primary variable of interest [3, 13, 14]. To ensure comparability between measurements, the most central slice in which the valve plane was visible was chosen for analysis. RV free wall endocardial contours were manually drawn during end-diastole and/or end-systole (as required by the individual software method) with subsequent automatic tracking during the cardiac cycle. As an example, a cine CMR movie file of the RV free wall endocardial tracking is available as Additional file 1. Endocardial tracking was visually evaluated and manually corrected if possible to ensure accurate tracking. Subsequently, the endocardial border was automatically segmented into three regions of equal size that were denoted subtricuspid, anterior, and apical wall (see Fig. 1), as previously described [13]. Global strain was defined as the average peak strain value across all segments.

#### Global RV size and ejection fraction

RV and left ventricular (LV) dimensions and function were measured with CVI42 (Circle Cardiovascular Imaging; Client Version 248, Server Version 258). Ventricular end-diastolic (EDV) and end-systolic volumes (ESV) were corrected for body surface area (BSA) according to the DuBois formula [16].

### Qualitative analysis

#### Feasibility

FT-CMR tracking quality of the endocardial border was visually assessed in each software method by one observer who was blinded for study group and demographic data. Segments in which FT tracking was obviously beyond the contours of the RV were excluded. To ensure consistency in the exclusion of segments, a second observer independently assessed a randomly selected subset of 40 patients.

#### Reproducibility

Intra-observer variability was evaluated by re-measuring RV peak strain in 40 randomly selected subjects by the first observer. For inter-observer variability, the same 40 subjects were measured by a second observer, independent from the first observer. Intra- and inter-observer variability was assessed for every software method separately. Observers were blinded for clinical and demographic data at the time of CMR measurements.

### Statistical analysis

Continuous and categorical variables are presented as mean (±standard deviation) and *n* (%), respectively. For continuous comparisons of two groups, two-tailed Student’s t-test was used (paired and unpaired as appropriate). For continuous comparisons of three or more groups, one-way ANOVA or Kruskall Wallis was used. Categorical data were compared using the chi-square test. A *p*-value of <0.05 was considered significant. Intra- and inter-observer reproducibility of strain measurements was evaluated visually by Bland-Altman analysis (MedCalc Software, version 16.8.4, Mariakerke, Belgium). Correlation was assessed by Intraclass Correlation Coefficient (ICC). For ICC, a value ≥0.75 was considered excellent, <0.75 and ≥0.40 moderate, and <0.40 poor. Diagnostic accuracy was evaluated using the area under the Receiver Operating Characteristic (ROC) curve. For the area under the curve (AUC), a value of 0.90–1.0 was considered excellent, 0.80–0.90 good, 0.70–0.80 moderate and <0.60 poor. Statistical analyses were performed using IBM SPSS statistics (IBM, version 21, Chicago, IL, USA).

## Results

### Baseline characteristics

We evaluated CMR images of 110 subjects including 39 (36%) overt ARVD/C patients (mutation+, phenotype+), 40 (36%) preclinical ARVD/C subjects (mutation+, phenotype-) and 31 (28%) healthy control subjects. Baseline characteristics of the study population are shown in Table 1. Overall, 56 (51%) subjects were male with a mean age of 33.3 ± 15.8 years. There were no significant differences between overt ARVD/C patients, preclinical ARVD/C patients, and control subjects in age (*p* = 0.341) and sex (*p* = 0.639). As expected, overt ARVD/C patients had higher RV EDV/BSA (88.3 ± 25.6 mL/m^3^) compared to preclinical (68.4 ± 14.4 mL/m^2^) and control subjects (69.7 ± 12.9 mL/m^2^)(*p* < 0.001 for trend). In addition, RV function was decreased in overt (48.3 ± 11.7%) compared to preclinical (54.9 ± 9.6%) and control subjects (56.9 ± 9.7%) (*p* = 0.005 for trend). LV volume and function did not differ between the groups (Table 1).

**Table 1:** Baseline characteristics of the study population

	OVERT ARVD/C (*N* = 39)	Preclinical ARVD/C (*n* = 40)	Controls (*n* = 31)
Female (%)	22 (56)	18 (45)	14 (45)
Age (yrs)	32.3 ± 13.5	31.3 ± 18.1	37.2 ± 14.9
Global CMR parameters			
RV EDV/BSA (mL/m^2^)	88.3 ± 25.6^a^	68.4 ± 14.4	69.7 ± 12.9
RV ESV/BSA (mL/m^2^)	47.4 ± 24.9^a^	31.1 ± 9.8	30.0 ± 8.6
RV EF (%)	48.3 ± 11.7^a^	54.9 ± 9.6	56.9 ± 9.7
LV EDV/BSA (mL/m^2^)	77.4 ± 12.1	69.1 ± 12.9	73.5 ± 9.6
LV ESV/BSA (mL/m^2^)	30.6 ± 9.5	25.2 ± 7.0	30.9 ± 15.1
LV EF (%)	62.7 ± 6.3	63.2 ± 11.7	58.9 ± 13.3
Clinical Phenotype			
Repolarization criteria			-
Major	36 (93)	1 (3)	
Minor	22 (54)	19 (48)	
Depolarization criteria			-
Major	3 (8)	0 (0)	
Minor	19 (49)	18 (45)	
Arrhythmia criteria			-
Major	7 (18)	0 (0)	
Minor	26 (67)	4 (10)	
Structural criteria			-
Major	16 (41)	0 (0)	
Minor	7 (18)	0 (0)	
TFC fulfillment: number of criteria (median)	6 (IQR 5–7)	2 (IQR 2–3)	-

*Abbreviations*: *ARVD/C* Arrhythmogenic Right Ventricular Dysplasia/Cardiomyopathy, *BSA* Body Surface Area, *EDV* End-Diastolic Volume, *EF* Ejection Fraction, *ESV* End-Systolic Volume, *TFC* Task Force Criteria, *N* number of subjects

^a^ Statistical significant difference compared to control subjects

### Feasibility comparison among software methods

**Fig. 2:**
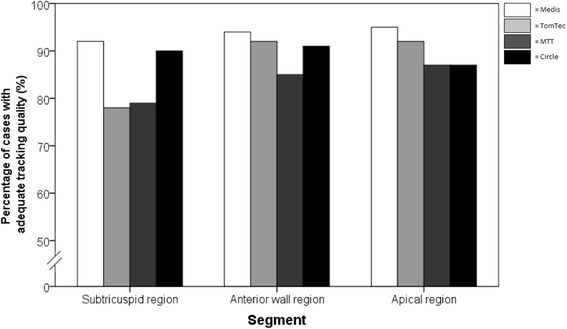
Percentage of cases with adequate tracking quality of the endocardial border stratified per segment and software method

We first performed a quality assessment to determine feasibility of strain measurements for every FT-CMR software method separately. Tracking quality was visually assessed for every study subject and dichotomized into adequate and inadequate tracking. Figure 2 shows the percentage of cases with adequate tracking quality stratified by segment and by software method. Zero subjects from Medis, 4 from TomTec, 7 from MTT and 9 from Circle were excluded in cases where the software would not read the image data. Of the remaining cases, tracking quality was highest in Medis (93% [308/330 segments of 95/110 subjects]), followed by Circle (89% [271/303 segments of 79/101 subjects]), TomTec (87% [277/318 segments of 80/106 subjects]) and MTT (84% [259/309 segments of 78/103 subjects included]). Furthermore, the tracking quality in the apical region (95%, 92%, 87%, and 87% for Medis, TomTec, MTT, and Circle respectively), anterior wall region (94%, 92%, 85%, and 91% for Medis, TomTec, MTT, and Circle respectively) and the subtricuspid region (92%, 78%, 79%, and 90% for Medis, TomTec, MTT, and Circle respectively) (Fig. 2) differed per software method. When stratifying by diagnostic group, the highest tracking quality was observed in preclinical ARVD/C (92% [429/468 segments]) and control subjects (91% [328/360 segments]), followed by overt ARVD/C patients (83% [365/438 segments]). Twenty percent of cases with low tracking quality showed overlap with at least one other software method. The minor overlap in cases of low tracking quality among software methods suggests that tracking quality is software-specific and not image quality- or patient-specific.

### FT-CMR intersoftware comparison of global and regional longitudinal strain

For the second part of our analyses, we excluded subjects with low tracking quality, since disturbed tracking will result in outliers that are not representative for actual wall motion of the included subjects. Analyses including all subjects regardless of tracking quality can be found in Additional files 2, 3, 4, and 5.

#### Global strain

**Fig. 3:**
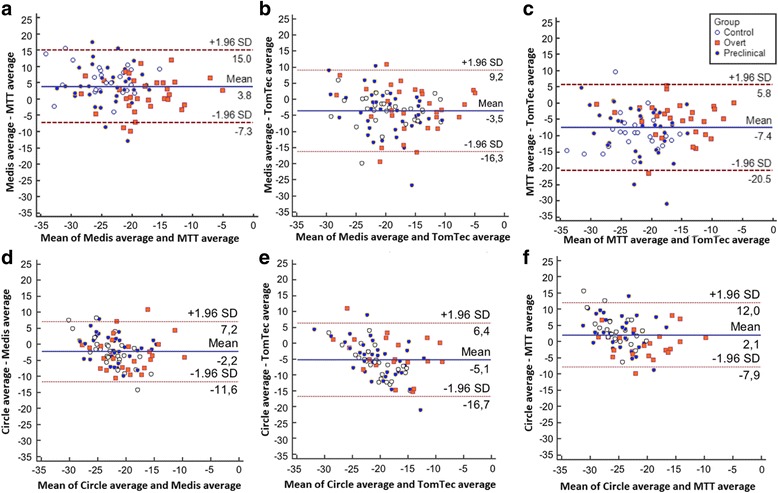
Bland-Altman plots per intersoftware variability of average right ventricular strain values. Intersoftware variability of strain values in **a**) Medis vs. MTT; **b**) Medis vs. TomTec; **c**) MTT vs. TomTec; **d**) Circle vs. Medis; **e**) Circle vs. TomTec; **f**) Circle vs. MTT

**Fig. 4:**
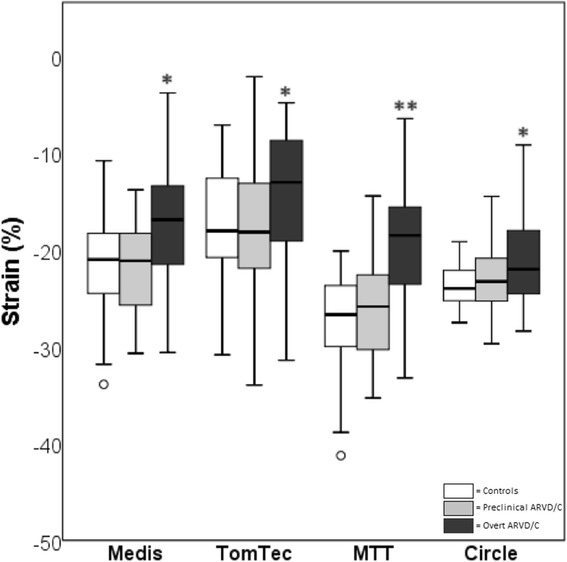
Global (average) strain by group per software package. Statistical significant difference compared to control subjects is expressed in * = *p* < 0.05 and ** = *p* < 0.01. Abbreviations: MTT = Multimodality Tissue Tracking

Table 2 shows global (average) peak strain for the four software methods stratified by ARVD/C diagnosis. While the magnitude of strain values was smaller (i.e. closer to zero) with TomTec than with the other three methods (*p* < 0.001 in the overall study population), all four methods showed a relative group difference with a trend towards lower strain values in overt ARVD/C patients compared to preclinical and control subjects. As shown in Fig. 3, Bland-Altman analyses with 95% limits of agreement shows a wide limit of agreement of >20% between the various software methods. This is also expressed by the ICC of 0.442 for absolute global strain values between the four software methods. In contrast, the distributions (standard deviations) of the average peak strain values were comparable between the different software methods, indicating that the spread of measurement is similar among software methods (Fig. 4).

**Table 2:** Right ventricular global (average) strain values stratified by diagnostic group^d^

	Overt ARVD/C (*N* = 39)	Preclinical ARVD/C (*N* = 40)	Controls (*N* = 31)	P-Value^c^
MEDIS	−17.6 ± 6.3^ab^	−21.8 ± 4.6	−21.4 ± 5.5	0.001
TOMTEC	−14.3 ± 7.1^ab^	−17.7 ± 6.6	−17.8 ± 5.6	0.057
MTT	−19.3 ± 6.2^ab^	−26.2 ± 5.0	−27.7 ± 5.5	<0.001
CIRCLE	−21.3 ± 5.3 ^a^	−22.9 ± 3.7	−23.7 ± 2.3	0.065

^a^ Statistical significant difference compared to control subjects; ^b^ = Statistical significant difference compared to preclinical subjects; ^c^ = Trend between overt ARVD/C patients, preclinical ARVD/C and control subjects (OneWay ANOVA). Abbreviations: ARVD/C = Arrhythmogenic Right Ventricular Dysplasia/ Cardiomyopathy; MTT = Multimodality Tissue Tracking; N = number of subjects

^d^ Segments included based on adequate tracking quality: 365/438, 429/468, and 328/360 segments in overt ARVD/C, preclinical ARVD/C and control subjects respectively

#### Regional strain

**Fig. 5:**
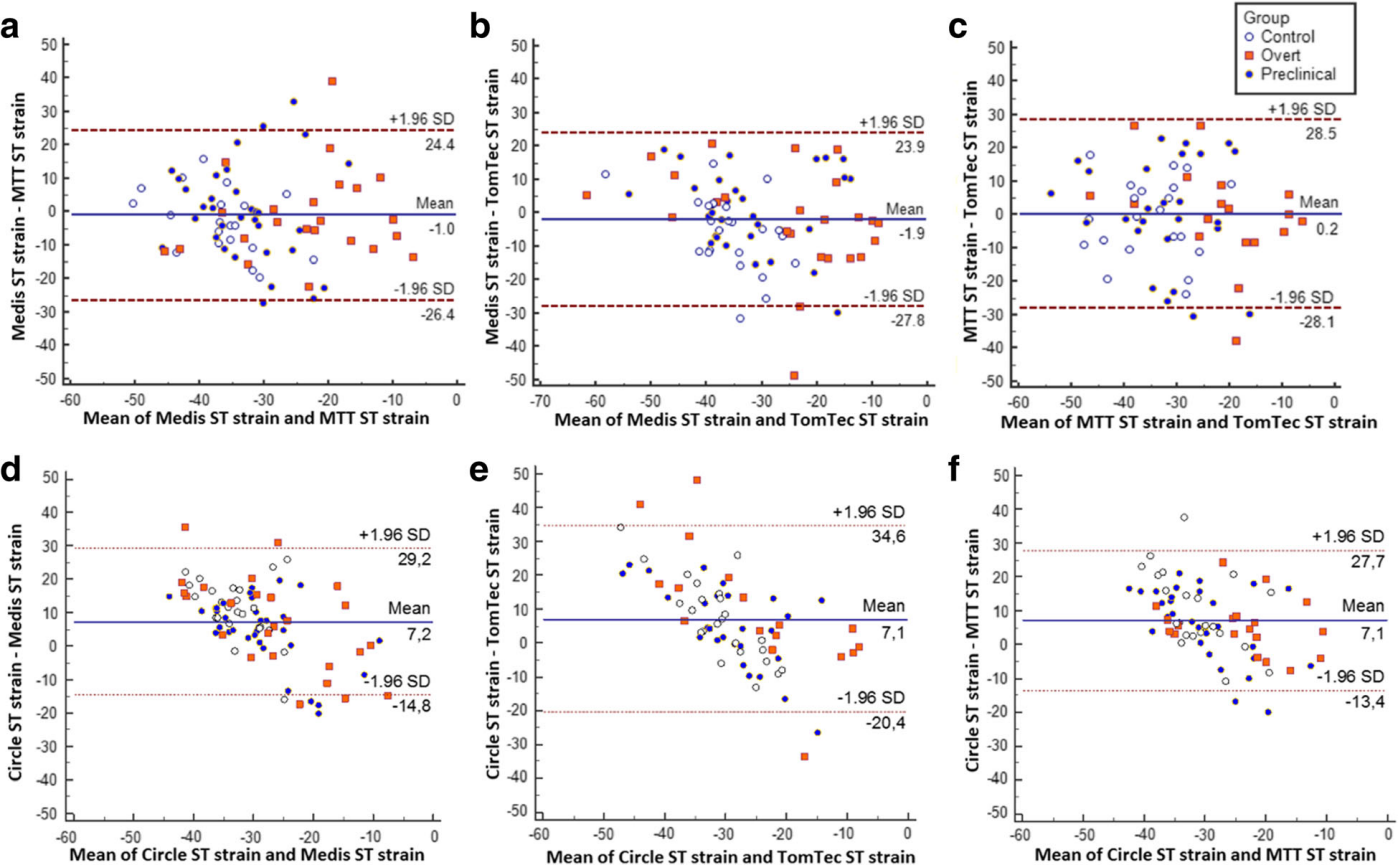
Bland-Altman plots per intersoftware variability of right ventricular subtricuspid strain values. Intersoftware variability of strain values in the subtricuspid region in **a**) Medis vs. MTT; **b**) Medis vs. TomTec; **c**) MTT vs. TomTec; **d**) Circle vs. Medis; **e**) Circle vs. TomTec; **f**) Circle vs. MTT. ST = subtricuspid region

**Fig. 6:**
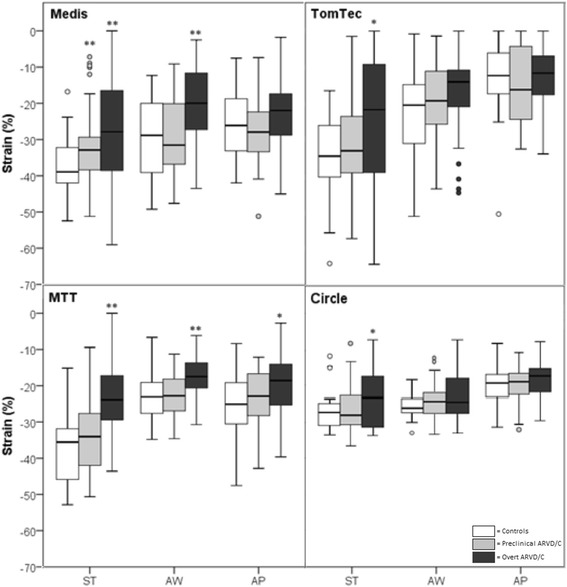
Regional strain by subgroup per software package. Statistical significant difference compared to control subjects is expressed in * = *p* < 0.05 and * = *p* < 0.01. Abbreviations: ST = subtricuspid region; AW = anterior wall region; AP = apical region; MTT = Multimodality Tissue Tracking

Table 3 shows regional (segmental) peak strain for the four software methods stratified by ARVD/C diagnosis. Again, the magnitude of the strain values in the anterior wall and the apical region was smaller (i.e. closer to zero) with TomTec compared to the other three methods (*p* < 0.001 in the overall study population). All four software methods showed a relative group difference with a trend towards lower strain in overt ARVD/C patients compared to preclinical and control subjects. As shown in Fig. 5, Bland-Altman analyses with 95% limits of agreement showed that there is moderate agreement between the software methods with wide limits of agreement for absolute subtricuspid strain values. This is also expressed by the ICC of 0.373 for absolute subtricuspid strain values between the four software methods. The distribution (standard deviation) of the segmental strain, especially in the subtricuspid region, was wider in TomTec than in other methods indicating a wider spread of measurements (Fig. 6). On the contrary, Circle showed a consistently lower distribution of the segmental strain and therefore a smaller spread of measurements.

**Table 3:** Right ventricular regional (segmental) strain values stratified by diagnostic group^d^

	Overt ARVD/C (*N* = 39)	Preclinical ARVD/C (*N* = 40)	Controls (*N* = 31)	P-Value^c^
Subtricuspid Region				
MEDIS	−-28.4 ± 14.8^a^	−31.6 ± 10.4^a^	−38.1 ± 8.1	0.007
TOMTEC	−24.7 ± 18.3^a^	−32.4 ± 12.6	−34.3 ± 11.4	0.045
MTT	−24.4 ± 10.8^ab^	−33.4 ± 10.9	−36.9 ± 10.5	<0.001
CIRCLE	−23.0 ± 8.4^a^	−26.2 ± 6.3	−27.0 ± 5.0	0.067
Anterior Wall Region				
MEDIS	−20.6 ± 10.5^ab^	−28.6 ± 10.3	−29.0 ± 11.1	0.001
TOMTEC	−17.5 ± 11.9	−19.7 ± 11.1	−22.6 ± 12.5	0.248
MTT	−17.7 ± 6.4 ^ab^	−23.0 ± 6.1	−22.8 ± 6.3	0.001
CIRCLE	−22.8 ± 6.2	−24.2 ± 5.0	−25.3 ± 3.4	0.168
Apical Region				
MEDIS	−22.8 ± 10.0^b^	−27.8 ± 8.7	−25.1 ± 9.6	0.072
TOMTEC	−12.7 ± 10.7	−14.7 ± 10.4	−13.1 ± 8.6	0.674
MTT	−18.6 ± 8.8^ab^	−23.3 ± 7.8	−25.5 ± 9.6	0.019
CIRCLE	−18.3 ± 5.4	−19.9 ± 5.1	−19.5 ± 5.6	0.521

^a^ Statistical significant difference compared to control subjects; ^b^ Statistical significant difference compared to preclinical subjects; ^c^ Trend between overt ARVD/C patients, preclinical ARVD/C and control subjects (OneWay ANOVA). Abbreviations: ARVD/C = Arrhythmogenic Right Ventricular Dysplasia/ Cardiomyopathy; MTT = Multimodality Tissue Tracking; N = number of subjects

^d^Segments included based on adequate tracking quality: 365/438, 429/468, and 328/360 segments in overt ARVD/C, preclinical ARVD/C and control subjects respectively

#### Reproducibility

As shown in Table 4, software methods showed moderate to excellent inter- and intra-observer reproducibility for the regional strain values, with inter-observer reproducibility ranging from 0.519 to 0.896 in the subtricuspid region, 0.677 to 0.864 in the anterior wall, and 0.472 to 0.861 in the apical wall. For all regions, the highest intra- observer reproducibility was seen in Circle (ICC ranging from 0.944 to 0.980), followed by Medis (ICC ranging from 0.909 to 0.954), TomTec (ICC ranging from 0.699 to 0.864), and MTT (ICC ranging from 0.696 to 0.806).

**Table 4:** Intra- and inter-observer reproducibility of regional (segmental) strain per software method

	Intra-Observer ICC	Inter-Observer ICC
Subtricuspid Region		
MEDIS	0.928	0.896
TOMTEC	0.816	0.538
MTT	0.696	0.519
CIRCLE	0.980	0.719
Anterior Wall Region		
MEDIS	0.954	0.792
TOMTEC	0.699	0.864
MTT	0.806	0.677
CIRCLE	0.969	0.783
Apical Region		
MEDIS	0.909	0.807
TOMTEC	0.790	0.861
MTT	0.787	0.472
CIRCLE	0.944	0.577

An ICC ≥0.75 was considered excellent, an ICC between <0.75 and ≥0.40 moderate, and an ICC <0.40 poor. Abbreviations: MTT = Multimodality Tissue Tracking; ICC = Intraclass Correlation Coefficient

### Clinical implementation of FT-CMR for early ARVD/C disease detection

With regards to global (average) strain (Fig. 4), overt patients had reduced strain compared to control subjects, which reached significance in all software methods (*p* < 0.041). In contrast, global strain was similar in preclinical and control subjects for all software methods (*p* > 0.275), suggesting that global strain is insensitive for early disease detection. With regards to regional (segmental) strain (Fig. 6), overt patients had reduced strain compared to control subjects, reaching statistical significance in the subtricuspid region for Medis, TomTec, MTT and Circle (*p* < 0.037), in the anterior wall for Medis and MTT (*p* < 0.005) and in the apex for MTT only (*p* = 0.012). While comparable regional strain values were observed for the anterior wall and apex, preclinical patients were separated from controls in the subtricuspid region by Medis software (*p* = 0.009). This is also illustrated by a moderate discriminative accuracy of subtricuspid strain to distinguish preclinical from control subjects using Medis (AUC = 0.70). For TomTec, MTT and Circle, preclinical patients cannot be discriminated from controls (AUC 0.53–0.58) using the subtricuspid region. Furthermore, the discriminative accuracy of FT-CMR in overt ARVD/C patients and control subjects was moderate to good in the subtricuspid region (AUC 0.64–0.80) and poor to moderate in both the anterior wall (AUC 0.61–0.74) and the apical wall region (AUC 0.47–68). AUC for global and regional longitudinal strain values in ARVD/C vs. control and preclinical vs. control can be found in Additional file 6.

## Discussion

Over the years, we have come to appreciate that ARVD/C starts as a regional rather than a global disease [9, 17]. This is reflected in the 2010 diagnostic TFC, which require the presence of regional RV wall motion abnormalities for fulfillment of diagnostic criteria [15]. Up to now, these wall motion abnormalities are assessed qualitatively and are thereby ‘in the eye of the beholder’ [18]. FT-CMR is a novel technique that may be useful for quantitative evaluation of regional RV strain. A challenge for clinical implementation is the absence of an independent standard of reference for RV strain values. The study of ARVD/C patients with FT techniques is advantageous in this regard, in that multiple independent criteria are used for diagnosis of the disease, including genetic testing, electrical abnormalities and family history. Our study aimed to assess intersoftware agreement of RV global and regional strain using FT-CMR. Our results show that significant variability exists between FT software methods, including 1) sporadic failure of RV wall tracking and 2) significant differences in absolute RV strain values. However, despite software variability, all four software methods were able to identify overt ARVD/C patients from control subjects on a group perspective using global strain. This may suggest some robustness of the FT-CMR approach. In addition, regional strain was reduced in overt ARVD/C patients compared to control subjects in all segments, which was most apparent in the subtricuspid region. Preclinical patients were distinguished from control subjects by decreased subtricuspid strain using one software method. These results suggest a role for FT-CMR in ARVD/C evaluation, pending further technological refinements.

### Overview of strain measurements of the right ventricle

Starting with the application of crystal sonomicrometry in dogs in the 1970s, the last decades have witnessed a surge in imaging techniques that can visualize local myocardial wall motion (deformation) [19]. Tissue tagging, a CMR technique that prescribes multiple grids on the myocardial tissue to track deformation throughout the cardiac cycle, is typically regarded as the gold standard for LV deformation [20]. Echocardiographic deformation imaging using either speckle tracking or tissue Doppler imaging has also gained popularity for those patients unfit to undergo CMR examinations [2, 21]. Of note, these techniques are technically demanding, time consuming, and have primarily been validated for use in the LV, but render themselves less suitable for the thin-walled and highly trabeculated RV [22]. In the context of these shortcomings, FT-CMR has been developed as an alternative for the assessment of both LV and RV strain. After the first publication on FT-CMR by Maret et al. [23], several research groups have confirmed its diagnostic value for LV evaluation [5, 24–27]. FT-CMR also gained popularity for assessment of the RV: it has been shown to be of clinical value in (repaired) tetralogy of Fallot [3, 28] and pulmonary hypertension [29, 30]. In addition, we and others have used FT-CMR in an ARVD/C population [12–14]. Additional file 7 provides an overview of global and regional strain values obtained in these prior ARVD/C populations. Heermann et al. showed that global RV strain values were significantly reduced in overt ARVD/C patients (*n* = 20) compared to healthy volunteers (*n* = 10) and family members (*n* = 22) [14]. Vigneault et al. (whose study population was identical to the present study) confirmed these findings and determined the horizontal longitudinal axis as the most reliable view to perform strain measurements [13]. Subsequently, Prati et al. showed that reduced global RV strain is present when global RV function is still preserved. While these results are promising, routine use of FT-CMR in clinical practice remains premature: FT-CMR needs to be standardized between software methods and RV wall tracking requires to be more reliably tracked [12]. Our study provides data addressing both these concerns.

### Feasibility of FT-CMR using different software methods

Our study provides a head-to-head comparison of four commercially available software methods for FT-CMR measurements of the RV. We show that feasibility of RV strain by FT-CMR is not uniform across software methods, and that absolute strain values correlate poorly with large limits of agreement. It therefore remains impossible to translate strain values obtained in one software method to another, at least on a patient-by-patient level. These findings are in line with previous studies using speckle tracking echocardiography, which showed poor correlation across software methods in healthy controls [31, 32]. Nagata et al. even showed significant variability of measurements using different versions of the same speckle tracking software [31]. The optimal performance of feature tracking relies on both algorithm-dependent and algorithm-independent properties [1]. As for algorithm-dependent properties, accuracy of feature tracking is determined by the interrogation window that determines the frame-to-frame tracking of the feature, the specific features which are extracted and the influence of other motions such as blood flow near the endocardial border [1, 33, 34]. As for algorithm-independent properties, image quality, the presence of trackable anatomic features and spatial/temporal resolution are likely essential determinants of accurate strain measurement [1, 35]. Our study is unique in the sense that it used the same CMR scans to test four software methods, so that the observed differences are due to an algorithm-dependent difference. Indeed, the subset of scans excluded based on low tracking quality (i.e. feasibility) was different for every software method, suggesting that tracking quality is algorithm-dependent. While it is expected that MTT, TomTec, Medis and Circle use different strain calculation algorithms, the low agreement between these methods is remarkable since tracking quality was determined to be adequate by two independent observers and patient-specific factors were constant by study design. Because no gold standard for RV strain exists, a normative comparison of the quality of these algorithms remains challenging [36]. Further refinements of the technique are necessary to increase comparability among software methods.

### Inter- and intra-observer reproducibility

All software methods showed a moderate to excellent inter- and intra-observer reproducibility, with higher intra-observer (ICC 0.69–0.98) compared to inter-observer (ICC 0.47–0.90) reproducibility. In general, Medis and Circle had higher inter- and intra-observer reproducibility than TomTec and MTT. Indeed, Medis and Circle showed higher tracking quality than the other two packages. Both TomTec and MTT required more manual adjustments of the endocardial contour. These manual adjustments may have influenced reproducibility, especially for inter-observer reproducibility. A difference between inter- and intra-observer reproducibility was also observed in previous studies [3, 12, 30, 37]. These studies all focused on the reproducibility of global strain, and all used the TomTec software method [3, 12, 30, 37, 38]. The inter-observer (ICC 0.61–0.75 and coefficient of variation (CV) 8.3–9.9%) and intra-observer (ICC 0.96–0.99 and CV 8.6–28.7%) reproducibility of these studies varied from moderate to excellent, which is comparable to our results [30, 35, 37, 38]. To the best of our knowledge, no previous studies investigated inter-and intra-observer reproducibility for RV regional strain. While the similarity between our results and prior observations is reassuring, it is important to note that especially inter-observer variability remains relatively poor for some of the software packages. Future refinement of the software algorithms will be necessary to further reduce variability between readers.

### Clinical value of global strain in ARVD/C evaluation

Despite the abovementioned intersoftware variability, our study shows that global strain is significantly reduced in overt ARVD/C patients compared to controls for all four software methods. This suggests that FT-CMR has the potential to differentiate healthy from diseased subjects, at least in group analysis. However, for an individual study subject, identification of global and regional strain abnormalities is variable, depends on image quality and varies between different software packages, limiting the ability to draw conclusions at an individual patient level. One could argue that differences in software variability are less visible for global strain measurements, since it provides a mean of all RV segments thereby averaging out measurement errors in a “trend towards the mean”. While these results are reassuring, the finding of lower global strain in overt ARVD/C patients may not be surprising, since global structural abnormalities are thought to occur late in the disease course of ARVD/C and are therefore expected to be abnormal at time of overt disease [17]. Indeed, RV ejection fraction by itself may be easier to implement and interpret compared to FT-CMR. Nevertheless, given the high degree of difficulty for interpretation of the RV, CMR physicians may be reassured by the finding of abnormal global RV strain in overt ARVD/C.

### Clinical value of regional strain in ARVD/C evaluation: Role for early disease detection?

Several studies have indicated that regional abnormalities occur prior to the onset of global changes in ARVD/C [17, 39]. As such, regional strain would be of particular interest as a tool for (early) diagnosis of this disease. Indeed, the results of our study show that regional strain is reduced in ARVD/C patients compared to controls, which is most consistent for the subtricuspid region. This is intriguing since abnormal subtricuspid strain has previously been shown (in a multivariable analysis controlling for gender, RV EF and RV EDV) to be an independent predictor for ARVD/C diagnosis, suggesting added value beyond RV size and function [13]. Furthermore, these results are also interesting in the context of our understanding of ARVD/C as a regional disease. In 1982, Marcus et al. described the “Triangle of Dysplasia” involving dyskinesia/aneurysms in the RV inferior wall (inflow tract), RV outflow tract, and RV apex in ARVD/C patients with a severe clinical phenotype [40]. More recently, we have come to appreciate that (mutation-positive) ARVD/C preferentially affects the subtricuspid region [9, 41–43], and spreads to the RV outflow tract and apex in later stages of disease [9]. Of note, subtricuspid strain was reduced in preclinical patients compared to control subjects for Medis software, suggesting a role for subtricuspid strain in early ARVD/C diagnosis. However, one should keep in mind that these results were obtained for one software method only, and should be validated in an external patient sample. It would be interesting for future studies to evaluate disease development over time in preclinical subjects with reduced subtricuspid strain and to investigate the value of FT-CMR in discriminating subjects with favorable and adverse clinical outcome.

### Limitations and perspective on clinical FT-CMR implementation

Our results highlight the potentially interesting role of FT-CMR for ARVD/C evaluation, but also indicate the need for further refinements in this technique. While the moderate to excellent reproducibility of FT-CMR renders this technique suitable for follow-up of ARVD/C patients, determination of the spectrum of normal RV strain values and thresholds for disease will help in standardization of FT-CMR. Evaluation of intersoftware variability of LV strain would be interesting but was beyond the scope of this study. Similar to the 2D speckle tracking-derived bull’s eye plots for LV longitudinal strain in cardiomyopathy patients, future FT-CMR studies on RV strain should consider incorporating a mapping for RV strain [44]. This may improve our understanding of RV strain distribution in health and disease. A limitation of this study is that we did not include a reference standard for RV strain. However, no validated reference standard for RV strain currently exists. Future studies should compare FT-CMR to other (CMR-based) deformation techniques to further optimize the FT technique. Regional feature tracking for strain calculation is likely dependent on algorithm-independent properties such as resolution and the presence of trackable anatomic features. Therefore, studies specifically investigating these properties such as the influence of spatial or temporal resolution will be helpful for further technical refinements of FT-CMR. Until that time, routine use of FT-CMR in ARVD/C evaluation should take place at an experienced center with experienced CMR readers.

## Conclusions

In this cohort of well-phenotyped ARVD/C patients and healthy controls, we performed FT-CMR to measure RV strain using four commercially available software methods. We demonstrate that intersoftware variability exists for both feasibility and absolute strain values. Regardless, all software methods are able to differentiate affected ARVD/C patients from controls by global strain, suggesting robustness of FT-CMR measures. In addition, we reveal that the subtricuspid region is an indicator region of ARVD/C, in which abnormal strain is visible in overt patients for all included software methods and prior to disease expression for one software method. These results highlight the potential of FT-CMR as an early diagnostic test in ARVD/C.

## Additional files

Additional file 1: Movie Clip; Example of RV free wall endocardial tracking (Circle Cardiovascular Imaging). (MOV 875 kb)

Additional file 2: Table S1. RV average strain values stratified by diagnostic group, without exclusions based on tracking quality. (DOCX 47 kb)

Additional file 3: Table S2. RV segmental strain values stratified by diagnostic group, without exclusions based on tracking quality. (DOCX 71 kb)

Additional file 4: Figure S1. Global (average) strain by group per software package without exclusions based on tracking quality. Statistical significant difference compared to control subjects expressed in * = *p* < 0.05 and ** = *p* < 0.01. Abbreviations: MTT = Multimodality Tissue Tracking. (PNG 68 kb)

Additional file 5: Figure S2. Regional strain by subgroup per software package without exclusions based on tracking quality. Statistical significant difference compared to control subjects expressed in * = *p* < 0.05 and ** = *p* < 0.01. Abbreviations: ST = subtricuspid region; AW = anterior wall region; AP = apical region; MTT = Multimodality Tissue Tracking. (PNG 119 kb)

Additional file 6: Table S3. AUC for global and regional longitudinal strain in ARVD/C vs. control and preclinical vs. control. (DOCX 49 kb)

Additional file 7: Table S4. Global and regional longitudinal strain values in previous studies in overt ARVD/C and preclinical subjects. (DOCX 53 kb)

## Abbreviations

ARVD/C: Arrhythmogenic Right Ventricular Dysplasia/Cardiomyopathy
BSA: Body Surface Area
CMR: Cardiovascular Magnetic Resonance imaging
EDV: End Diastolic Volume
ESV: End Systolic Volume
FT-CMR: Feature Tracking Cardiovascular Magnetic Resonance
HLA: Horizontal Longitudinal Strain
ICC: Intraclass Correlation Coefficient
LV: Left Ventricle
MTT: Multimodality Tissue Tracking
RV: Right Ventricle
TFC: Task Force Criteria

## References

[1] Pedrizzetti G, Claus P, Kilner PJ, Nagel E (2016). Principles of cardiovascular magnetic resonance feature tracking and echocardiographic speckle tracking for informed clinical use. J Cardiovasc Magn Reson.

[2] Claus P, Omar AM, Pedrizzetti G, Sengupta PP, Nagel E (2015). Tissue tracking Technology for Assessing Cardiac Mechanics: principles, normal values, and clinical applications. JACC Cardiovasc Imaging..

[3] Kempny A, Fernandez-Jimenez R, Orwat S, Schuler P, Bunck AC, Maintz D (2012). Quantification of biventricular myocardial function using cardiac magnetic resonance feature tracking, endocardial border delineation and echocardiographic speckle tracking in patients with repaired tetralogy of Fallot and healthy controls. J Cardiovasc Magn Reson.

[4] Shehata ML, Cheng S, Osman NF, Bluemke DA, Lima JA (2009). Myocardial tissue tagging with cardiovascular magnetic resonance. J Cardiovasc Magn Reson.

[5] Augustine D, Lewandowski AJ, Lazdam M, Rai A, Francis J, Myerson S (2013). Global and regional left ventricular myocardial deformation measures by magnetic resonance feature tracking in healthy volunteers: comparison with tagging and relevance of gender. J Cardiovasc Magn Reson.

[6] Te Riele AS, Tandri H, Bluemke DA (2014). Arrhythmogenic right ventricular cardiomyopathy (ARVC): cardiovascular magnetic resonance update. J Cardiovasc Magn Reson.

[7] Dalal D, Nasir K, Bomma C, Prakasa K, Tandri H, Piccini J (2005). Arrhythmogenic right ventricular dysplasia: a United States experience. Circulation.

[8] van der Werf C, Hofman N, Tan HL, van Dessel PF, Alders M, van der Wal AC (2010). Diagnostic yield in sudden unexplained death and aborted cardiac arrest in the young: the experience of a tertiary referral center in The Netherlands. Heart Rhythm.

[9] Te Riele AS, James CA, Philips B, Rastegar N, Bhonsale A, Groeneweg JA (2013). Mutation-positive arrhythmogenic right ventricular dysplasia/cardiomyopathy: the triangle of dysplasia displaced. J Cardiovasc Electrophysiol.

[10] Aneq MA, Engvall J, Brudin L, Nylander E (2012). Evaluation of right and left ventricular function using speckle tracking echocardiography in patients with arrhythmogenic right ventricular cardiomyopathy and their first degree relatives. Cardiovasc Ultrasound..

[11] Prakasa KR, Wang J, Tandri H, Dalal D, Bomma C, Chojnowski R (2007). Utility of tissue Doppler and strain echocardiography in arrhythmogenic right ventricular dysplasia/cardiomyopathy. Am J Cardiol.

[12] Prati G, Vitrella G, Allocca G, Muser D, Buttignoni SC, Piccoli G (2015). Right ventricular strain and Dyssynchrony assessment in Arrhythmogenic right ventricular Cardiomyopathy: cardiac magnetic resonance feature-tracking study. Circ Cardiovasc Imaging.

[13] Vigneault DM, te Riele AS, James CA, Zimmerman SL, Selwaness M, Murray B (2016). Right ventricular strain by MR quantitatively identifies regional dysfunction in patients with arrhythmogenic right ventricular cardiomyopathy. J Magn Reson Imaging.

[14] Heermann P, Hedderich DM, Paul M, Schulke C, Kroeger JR, Baessler B (2014). Biventricular myocardial strain analysis in patients with arrhythmogenic right ventricular cardiomyopathy (ARVC) using cardiovascular magnetic resonance feature tracking. J Cardiovasc Magn Reson.

[15] Marcus FI, McKenna WJ, Sherrill D, Basso C, Bauce B, Bluemke DA (2010). Diagnosis of arrhythmogenic right ventricular cardiomyopathy/dysplasia: proposed modification of the task force criteria. Circulation.

[16] Du Bois D, Du Bois EF (1989). A formula to estimate the approximate surface area if height and weight be known. 1916. Nutrition.

[17] Te Riele AS, Tandri H, Sanborn DM, Bluemke DA (2015). Noninvasive multimodality imaging in ARVD/C. JACC Cardiovasc Imaging..

[18] Bluemke DA (2011). ARVC: imaging diagnosis is still in the eye of the beholder. JACC Cardiovasc Imaging.

[19] Carlsson E, Milne EN (1967). Permanent implantation of endocardial tantalum screws: a new technique for functional studies of the heart in the experimental animal. J Can Assoc Radiol.

[20] Wu L, Germans T, Guclu A, Heymans MW, Allaart CP, van Rossum AC (2014). Feature tracking compared with tissue tagging measurements of segmental strain by cardiovascular magnetic resonance. J Cardiovasc Magn Reson.

[21] Abraham TP, Dimaano VL, Liang HY (2007). Role of tissue Doppler and strain echocardiography in current clinical practice. Circulation.

[22] Mertens LL, Friedberg MK (2010). Imaging the right ventricle--current state of the art. Nat Rev Cardiol.

[23] Maret E, Todt T, Brudin L, Nylander E, Swahn E, Ohlsson JL (2009). Functional measurements based on feature tracking of cine magnetic resonance images identify left ventricular segments with myocardial scar. Cardiovasc Ultrasound.

[24] Schneeweis C, Qiu J, Schnackenburg B, Berger A, Kelle S, Fleck E (2014). Value of additional strain analysis with feature tracking in dobutamine stress cardiovascular magnetic resonance for detecting coronary artery disease. J Cardiovasc Magn Reson.

[25] Schuster A, Hor KN, Kowallick JT, Beerbaum P, Kutty S. Cardiovascular Magnetic Resonance Myocardial Feature Tracking: Concepts and Clinical Applications. Circ Cardiovasc Imaging. 2016;9:e004077. 10.1161/CIRCIMAGING.115.004077 27009468

[26] Buss SJ, Breuninger K, Lehrke S, Voss A, Galuschky C, Lossnitzer D (2015). Assessment of myocardial deformation with cardiac magnetic resonance strain imaging improves risk stratification in patients with dilated cardiomyopathy. Eur Heart J Cardiovasc Imaging..

[27] Onishi T, Saha SK, Ludwig DR, Onishi T, Marek JJ, Cavalcante JL (2013). Feature tracking measurement of dyssynchrony from cardiovascular magnetic resonance cine acquisitions: comparison with echocardiographic speckle tracking. J Cardiovasc Magn Reson.

[28] Jing L, Haggerty CM, Suever JD, Alhadad S, Prakash A, Cecchin F (2014). Patients with repaired tetralogy of Fallot suffer from intra- and inter-ventricular cardiac dyssynchrony: a cardiac magnetic resonance study. Eur Heart J Cardiovasc Imaging.

[29] Ohyama Y, Ambale-Venkatesh B, Chamera E, Shehata ML, Corona-Villalobos CP, Zimmerman SL (2015). Comparison of strain measurement from multimodality tissue tracking with strain-encoding MRI and harmonic phase MRI in pulmonary hypertension. Int J Cardiol.

[30] De Siqueira MEM, Pozo E, Fernandes VR, Sengupta PP, Modesto K, Gupta SS (2016). Characterization and clinical significance of right ventricular mechanics in pulmonary hypertension evaluated with cardiovascular magnetic resonance feature tracking. J Cardiovasc Magn Reson.

[31] Nagata Y, Takeuchi M, Mizukoshi K, Wu VC, Lin FC, Negishi K (2015). Intervendor variability of two-dimensional strain using vendor-specific and vendor-independent software. J Am Soc Echocardiogr.

[32] Farsalinos KE, Daraban AM, Unlu S, Thomas JD, Badano LP, Voigt JU (2015). Head-to-head comparison of global longitudinal strain measurements among nine different vendors: the EACVI/ASE inter-vendor comparison study. J Am Soc Echocardiogr.

[33] Barron JL, Fleet DJ, Beauchemin SS (1994). Performance of optical flow techniques. Int J Comput Vis.

[34] Miozzi M, Jacob B, Olivieri A (2008). Performances of feature tracking in turbulent boundary layer investigation. Exp Fluids.

[35] Schuster A, Morton G, Hussain ST, Jogiya R, Kutty S, Asrress KN (2013). The intra-observer reproducibility of cardiovascular magnetic resonance myocardial feature tracking strain assessment is independent of field strength. Eur J Radiol.

[36] Tandri H, Castillo E, Ferrari VA (2006). Magnetic resonance imaging of arrhythmogenic right ventricular dysplasia. Sensitivity, specificity, and observer variability of fat detection versus functional analysis of the right ventricle. J Am Coll Cardiol.

[37] Schmidt B, Dick A, Treutlein M, Schiller P, Bunck AC (2017). Intra-and inter-observer reproducibility of global and regional magnetic resonance feature tracking derived strain parameters of the left and right ventricle. Eur J Radiol.

[38] Orwat S, Kempny A, Diller GP, Bauerschmitz P, Bunck AC (2014). Cardiac magnetic resonance feature tracking: a novel method of assessing myocardial strain. Comparison with echocardiographic speckle tracking in healthy volunteers and in patients with left ventricular hypertrophy. Kardiol Pol.

[39] Bomma C, Dalal D, Tandri H, Prakasa K, Nasir K, Roguin A (2005). Regional differences in systolic and diastolic function in arrhythmogenic right ventricular dysplasia/cardiomyopathy using magnetic resonance imaging. Am J Cardiol.

[40] Marcus FI, Fontaine GH, Guiraudon G, Frank R, Laurenceau JL, Malergue C (1982). Right ventricular dysplasia: a report of 24 adult cases. Circulation.

[41] Marchlinski FE, Zado E, Dixit S, Gerstenfeld E, Callans DJ, Hsia H (2004). Electroanatomic substrate and outcome of catheter ablative therapy for ventricular tachycardia in setting of right ventricular cardiomyopathy. Circulation.

[42] Bomma C, Dalal D, Tandri H, Prakasa K, Nasir K, Roguin A (2007). Evolving role of multidetector computed tomography in evaluation of arrhythmogenic right ventricular dysplasia/cardiomyopathy. Am J Cardiol.

[43] Marra MP, Leoni L, Bauce B, Corbetti F, Zorzi A, Migliore F (2012). Imaging study of ventricular scar in arrhythmogenic right ventricular cardiomyopathy: comparison of 3D standard electroanatomical voltage mapping and contrast-enhanced cardiac magnetic resonance. Circ Arrhythm Electrophysiol.

[44] Liu D, Hu K, Nordbeck P, Ertl G, Störk S, Weidemann F (2016). Longitudinal strain bull’s eye plot patterns in patients with cardiomyopathy and concentric left ventricular hypertrophy. Eur J Med Res.

