# Supplementary material for: Feature tracking CMR reveals abnormal strain in preclinical arrhythmogenic right ventricular dysplasia/ cardiomyopathy: a multisoftware feasibility and clinical implementation study
Source: J Cardiovasc Magn Reson. 2017 Sep 1;19:66. doi: 10.1186/s12968-017-0380-4 (PMC5581480; doi:10.1186/s12968-017-0380-4)
Supplement: Supplementary file 2 — RV average strain values stratified by diagnostic group, without exclusions based on tracking quality. (DOCX 47 kb) [file 12968_2017_380_MOESM2_ESM.docx]

**Additional File 1: Table 1; Right ventricular average strain values stratified by diagnostic group, without exclusions based on tracking quality**

|  | **OVERT ARVD/C (N=39)** | **PRECLINICAL ARVD/C (N=40)** | **CONTROLS**  **(N=31)** | **P-VALUE^#^** |
| --- | --- | --- | --- | --- |
|  |  | | | |
| MEDIS | -17.6 ± 6.1*^ | -21.9 ± 4.6 | -21.1 ± 5.6 | **0.002** |
| TOMTEC | -14.2 ± 7.0*^ | -17.7 ± 6.6 | -17.8 ± 5.6 | **0.043** |
| MTT  CIRCLE | -18.7 ± 6.2*^  -19.4 ± 5.9*^ | -25.4 ± 5.7  -22.6 ± 3.9 | -25.8 ± 7.2  -23.7 ± 2.3 | **<0.001**  **<0.001** |

*= Statistical significant difference compared to control subjects; ^= Statistical significant difference compared to preclinical subjects; #=Trend between overt ARVD/C patients, preclinical ARVD/C and control subjects (OneWay ANOVA). Abbreviations: MTT= Multimodality Tissue Tracking.
