# Supplementary material for: Feature tracking CMR reveals abnormal strain in preclinical arrhythmogenic right ventricular dysplasia/ cardiomyopathy: a multisoftware feasibility and clinical implementation study
Source: J Cardiovasc Magn Reson. 2017 Sep 1;19:66. doi: 10.1186/s12968-017-0380-4 (PMC5581480; doi:10.1186/s12968-017-0380-4)
Supplement: Supplementary file 3 — RV segmental strain values stratified by diagnostic group, without exclusions based on tracking quality. (DOCX 71 kb) [file 12968_2017_380_MOESM3_ESM.docx]

**Additional File 2: Table 2; Right ventricular segmental strain values stratified by diagnostic group, without exclusions based on tracking quality**

|  | **OVERT ARVD/C (N=39)** | **PRECLINICAL ARVD/C (N=40)** | **CONTROLS**  **(N=31)** | **P-VALUE^#^** | |
| --- | --- | --- | --- | --- | --- |
| **SUBTRICUSPID REGION** |  | | | |  |
| MEDIS | -29.3 ± 14.4* | -31.6 ± 10.3* | -36.6 ± 9.5 | **0.038** | |
| TOMTEC | -27.7 ± 18.8 | -31.8 ± 14.3 | -34.1 ± 13.5 | 0.247 | |
| MTT  CIRCLE | -25.5 ± 12.9*^  -21.3 ± 8.5*^ | -32.8 ± 10.5  -25.6 ± 6.5 | -36.0 ± 11.7  -25.9 ± 5.9 | **0.001**  **0.014** | |
| **ANTERIOR WALL REGION** |  |  |  |  | |
| MEDIS | -21.4 ± 10.8*^ | -28.4 ± 10.0 | -28.51 ± 11.2 | **0.005** | |
| TOMTEC | -16.1 ± 11.9* | -19.73 ± 11.1 | -23.3 ± 12.8 | 0.051 | |
| MTT  CIRCLE | -18.2 ± 6.6^  -21.4 ± 6.7* | -23.3 ± 7.3  -23.6 ± 5.5 | -20.9 ± 7.8  -25.3 ± 3.4 | **0.010**  **0.019** | |
| **APICAL REGION** |  |  |  |  | |
| MEDIS | -23.1 ± 10.0^ | -27.5 ± 8.7 | -25.1 ± 9.3 | 0.114 | |
| TOMTEC | -12.5 ± 8.6 | -14.7 ± 10.4 | -12.6 ± 10.2 | 0.540 | |
| MTT  CIRCLE | -18.3 ± 9.1*^  -15.6 ± 6.0* | -22.4 ± 8.3  -18.3 ± 5.2 | -23.7 ± 11.2  -19.1 ± 5.9 | 0.053  **0.038** | |

*= Statistical significant difference compared to control subjects; ^= Statistical significant difference compared to preclinical subjects; #= Trend between overt ARVD/C patients, preclinical ARVD/C and control subjects (OneWay ANOVA). Abbreviations: ARVD/C= Arrhythmogenic Right Ventricular Dysplasia/ Cardiomyopathy; MTT= Multimodality Tissue Tracking.
