# Supplementary figures and images for: Feature tracking CMR reveals abnormal strain in preclinical arrhythmogenic right ventricular dysplasia/ cardiomyopathy: a multisoftware feasibility and clinical implementation study
Source: J Cardiovasc Magn Reson. 2017 Sep 1;19:66. doi: 10.1186/s12968-017-0380-4 (PMC5581480; doi:10.1186/s12968-017-0380-4)

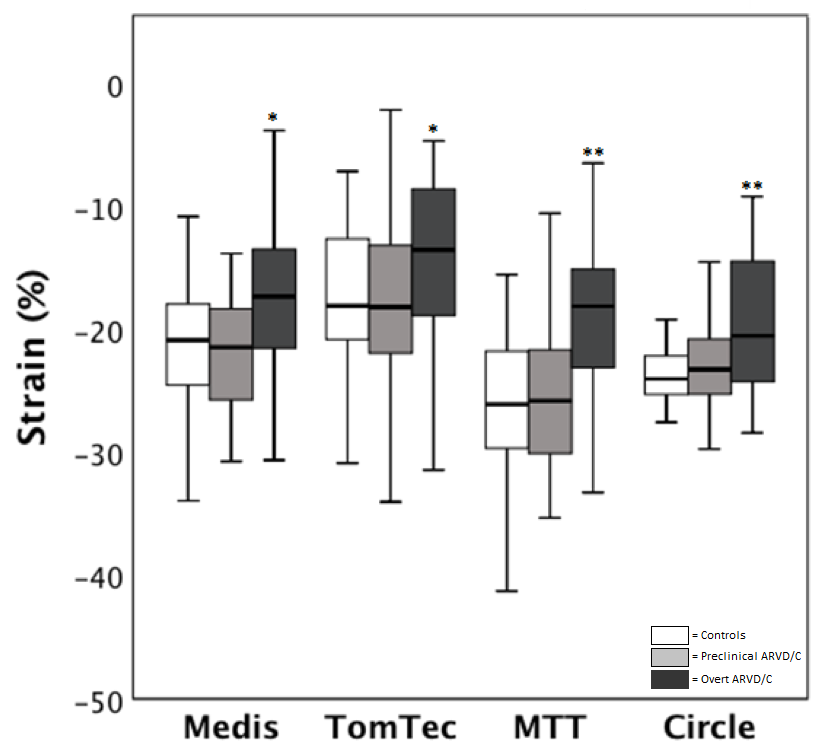

Supplement: Supplementary file 4 — Global (average) strain by group per software package without exclusions based on tracking quality. Statistical significant difference compared to control subjects expressed in * = p < 0.05 and ** = p < 0.01. Abbreviations: MTT = Multimodality Tissue Tracking. (PNG 68 kb) [file 12968_2017_380_MOESM4_ESM.png]

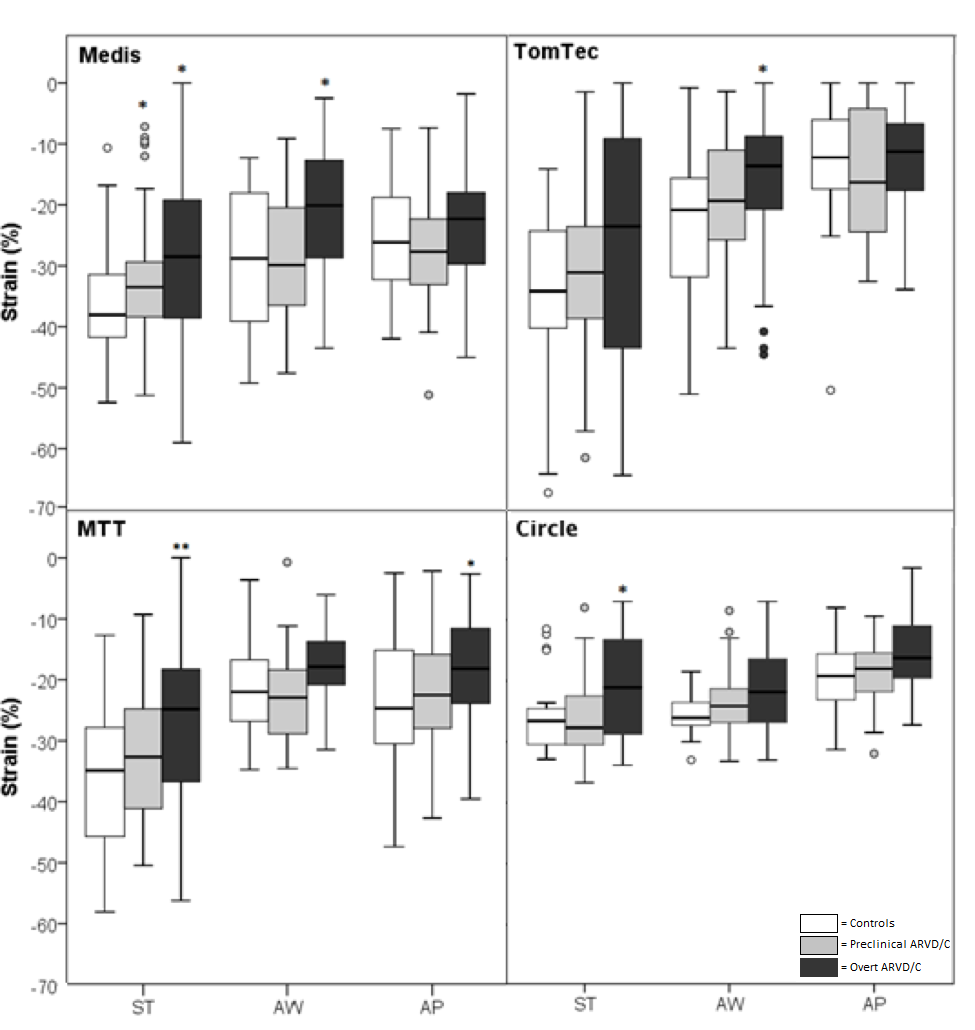

Supplement: Supplementary file 5 — Regional strain by subgroup per software package without exclusions based on tracking quality. Statistical significant difference compared to control subjects expressed in * = p < 0.05 and ** = p < 0.01. Abbreviations: ST = subtricuspid region; AW = anterior wall region; AP = apical region; MTT = Multimodality Tissue Tracking. (PNG 119 kb) [file 12968_2017_380_MOESM5_ESM.png]
