# Supplementary material for: Feature tracking CMR reveals abnormal strain in preclinical arrhythmogenic right ventricular dysplasia/ cardiomyopathy: a multisoftware feasibility and clinical implementation study
Source: J Cardiovasc Magn Reson. 2017 Sep 1;19:66. doi: 10.1186/s12968-017-0380-4 (PMC5581480; doi:10.1186/s12968-017-0380-4)
Supplement: Supplementary file 6 — AUC for global and regional longitudinal strain in ARVD/C vs. control and preclinical vs. control. (DOCX 49 kb) [file 12968_2017_380_MOESM6_ESM.docx]

**Additional File 6: Table 4; AUC for global and regional longitudinal strain values in overt ARVD/C vs. control and preclinical vs. control**

|  | **OVERT ARVD/C VS. CONTROL** | | | | **PRECLINICAL VS. CONTROL** | | | |
| --- | --- | --- | --- | --- | --- | --- | --- | --- |
|  | Medis | TomTec | MTT | Circle | Medis | TomTec | MTT | Circle |
| **GLOBAL STRAIN** | 0.67 | 0.67 | 0.86 | 0.63 | 0.48 | 0.50 | 0.56 | 0.57 |
|  |  |  |  |  |  |  |  |  |
| **REGIONAL STRAIN**  Subtricuspid  Anterior wall  Apex | 0.72  0.71  0.57 | 0.70  0.64  0.47 | 0.80  0.74  0.68 | 0.64  0.61  0.58 | 0.70  0.50  0.43 | 0.53  0.56  0.42 | 0.58  0.51  0.56 | 0.53  0.57  0.49 |

Abbreviations: AUC= Area under the (ROC) curve; ARVD/C= Arrhythmogenic Right Ventricular Dysplasia/ Cardiomyopathy; MTT= Multimodality Tissue Tracking.
