# Supplementary material for: Feature tracking CMR reveals abnormal strain in preclinical arrhythmogenic right ventricular dysplasia/ cardiomyopathy: a multisoftware feasibility and clinical implementation study
Source: J Cardiovasc Magn Reson. 2017 Sep 1;19:66. doi: 10.1186/s12968-017-0380-4 (PMC5581480; doi:10.1186/s12968-017-0380-4)
Supplement: Supplementary file 7 — Global and regional longitudinal strain values in previous studies in overt ARVD/C and preclinical subjects. (DOCX 53 kb) [file 12968_2017_380_MOESM7_ESM.docx]

**Additional File 5: Table 3; Global and regional longitudinal strain values in previous studies in overt ARVD/C and preclinical subjects**

| **ARTICLE (SOFTWARE)** | **GLOBAL** | **SUBTRICUSPID REGION** | **ANTERIOR WALL REGION** | **APICAL REGION** |
| --- | --- | --- | --- | --- |
| **OVERT ARVD/C PATIENTS** | | | | |
| VIGNEAULT ET AL.^13^ (MTT) | -19.3±6.2 | -24.4±10.8 | -17.7±6.4 | -18.6±8.8 |
| HEERMANN ET AL.^14^ (TomTec) | -12.7±7.3 | - | - | - |
| PRATI ET AL.^12^ (TomTec) | -17±5 | -22±11 | -15±8 | -14±8 |
| **PRECLINICAL ARVD/C SUBJECTS** | | | | |
| VIGNEAULT ET AL.^13^ (MTT) | -26.2±5.0 | -33.4±10.9 | -23.0±6.1 | -23.3±7.8 |
| HEERMANN ET AL.^14^ (TomTec) | -20.4±4.8 | - | - | - |

Abbreviations: ARVD/C= Arrhythmogenic Right Ventricular Dysplasia/ Cardiomyopathy; MTT= Multimodality Tissue Tracking.
